# Chromosome Engineering Allows the Efficient Isolation of Vertebrate Neocentromeres

**DOI:** 10.1016/j.devcel.2013.02.009

**Published:** 2013-03-25

**Authors:** Wei-Hao Shang, Tetsuya Hori, Nuno M.C. Martins, Atsushi Toyoda, Sadahiko Misu, Norikazu Monma, Ichiro Hiratani, Kazuhiro Maeshima, Kazuho Ikeo, Asao Fujiyama, Hiroshi Kimura, William C. Earnshaw, Tatsuo Fukagawa

**Affiliations:** 1 Department of Molecular Genetics, National Institute of Genetics and The Graduate University for Advanced Studies (SOKENDAI), Mishima, Shizuoka 411-8540, Japan; 2 Comparative Genomics Laboratory, National Institute of Genetics and The Graduate University for Advanced Studies (SOKENDAI), Mishima, Shizuoka 411-8540, Japan; 3 Cell Innovation Project, National Institute of Genetics and The Graduate University for Advanced Studies (SOKENDAI), Mishima, Shizuoka 411-8540, Japan; 4 Laboratory of Biological Macromolecules, National Institute of Genetics and The Graduate University for Advanced Studies (SOKENDAI), Mishima, Shizuoka 411-8540, Japan; 5 Wellcome Trust Centre for Cell Biology, University of Edinburgh, King’s Buildings, Mayfield Road, Edinburgh, EH9 3JR, UK; 6 National Institute of Informatics, Hitotsubashi, Chiyoda-ku, Tokyo 101-8430, Japan; 7 Graduate School of Frontier Biosciences, Osaka University, 1-3 Yamada-oka, Suita, Osaka 565-0871, Japan

## Abstract

Centromeres are specified by sequence-independent epigenetic mechanisms in most organisms. Rarely, centromere repositioning results in neocentromere formation at ectopic sites. However, the mechanisms governing how and where neocentromeres form are unknown. Here, we established a chromosome-engineering system in chicken DT40 cells that allowed us to efficiently isolate neocentromere-containing chromosomes. Neocentromeres appear to be structurally and functionally equivalent to native centromeres. Chromatin immunoprecipitation sequencing (ChIP-seq) analysis with 18 neocentromeres revealed that the centromere-specific histone H3 variant CENP-A occupies an ∼40 kb region at each neocentromere, which has no preference for specific DNA sequence motifs. Furthermore, we found that neocentromeres were not associated with histone modifications H3K9me3, H3K4me2, and H3K36me3 or with early replication timing. Importantly, low but significant levels of CENP-A are detected around endogenous centromeres, which are capable of seeding neocentromere assembly if the centromere core is removed. In summary, our experimental system provides valuable insights for understanding how neocentromeres form.

## Graphical abstract

**Graphical Abstract:**

**Figure:**
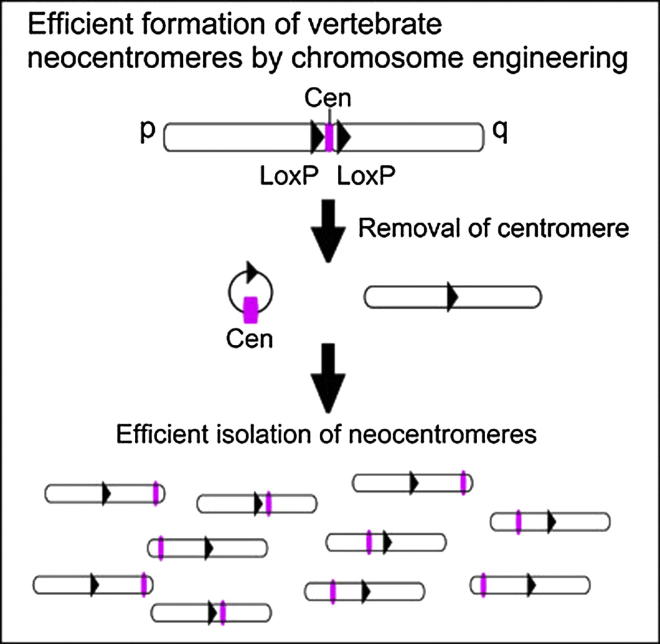

**Highlights:**

► Chromosome engineering efficiently generates neocentromeres in chicken DT40 cells ► CENP-A reproducibly occupies an ∼40 kb genomic region at each neocentromere ► Nonkinetochore CENP-A appears to function as a seed for neocentromere assembly

## Teaser

Centromeres are specified by sequence-independent epigenetic mechanisms. Shang et al. generated a collection of chicken neocentromeres in DT40 cells. Their analysis indicates that neocentromere formation does not correlate with the expected histone modifications or with replication timing, but rather depends on the histone H3 variant CENP-A to seed assembly.

## Introduction

The centromere is the genomic locus that directs faithful chromosome segregation. In human cells, the ability of cells to inactivate a centromere on dicentric chromosomes (Earnshaw and Migeon, 1985) and the formation of neocentromeres at unique DNA sequences lacking the alpha-satellite repeats traditionally associated with centromeres (du Sart et al., 1997; Marshall et al., 2008) together reveal that the underlying DNA sequence is neither necessary nor sufficient to specify centromere formation in vertebrate cells. It is now believed that centromeres are specified by sequence-independent epigenetic mechanisms involving the deposition of the centromere-specific histone H3 variant CENP-A (Allshire and Karpen, 2008; Black and Cleveland, 2011; Perpelescu and Fukagawa, 2011). However, as the rare neocentromeres observed in human patients allow only observational or correlative studies, and human neocentromeres are typically observed in adults after large numbers of cell generations have passed, little is known about the molecular events that lead to neocentromere formation. In *Schizosaccharomyces pombe* or *Candida albicans*, centromere deletion has been used to drive neocentromere formation, and the yeast model systems are widely used to understand molecular mechanisms for centromere formation (Ishii et al., 2008; Ketel et al., 2009). However, these fungal genomes are compact and contain few noncoding regions and repetitive sequences, and it is still unclear how neocentromeres form following centromere inactivation in vertebrate cells.

Here, we established a chromosome engineering system to efficiently generate neocentromeres in chicken DT40 cells. We previously demonstrated that at least three chicken chromosomes (chromosomes 5, 27, and Z) contain centromere loci of 30–40 kb lacking tandem repetitive sequences (Shang et al., 2010). In contrast, with the exception of the horse, which has one centromere on unique sequence DNA (Wade et al., 2009), centromeres of other vertebrates including humans typically encompass megabase (Mb) domains of complex repetitive sequences (Rudd et al., 2003), although the size of the CENP-A domain within the tandem repetitive region can vary widely (Sullivan et al., 2011). Chromosome engineering allowed us to conditionally excise the centromere on the chicken chromosome Z or 5. Here, we adapted this system to enable the efficient isolation of neocentromeres in DT40 cell lines, thus providing a resource to understand mechanisms of centromere formation. We isolated over 100 independent neocentromere-containing clones and mapped the distribution of CENP-A on 18 of them in detail by chromatin immunoprecipitation sequencing (ChIP-seq). Characterization of these multiple independent neocentromeres reveals that they encompass ∼40 kb of CENP-A-containing chromatin, but they were not consistently associated with histone modifications H3K9me3, H3K4me2 and H3K36me3 or with early replication timing. Interestingly, we found that low but significant levels of nonkinetochore CENP-A scattered throughout chromatin flanking the 40 kb centromeric CENP-A domain may seed for neocentromere formation following removal of the original centromere.

## Results

### Efficient Formation of Neocentromeres in DT40 Cells

**Figure 1:**
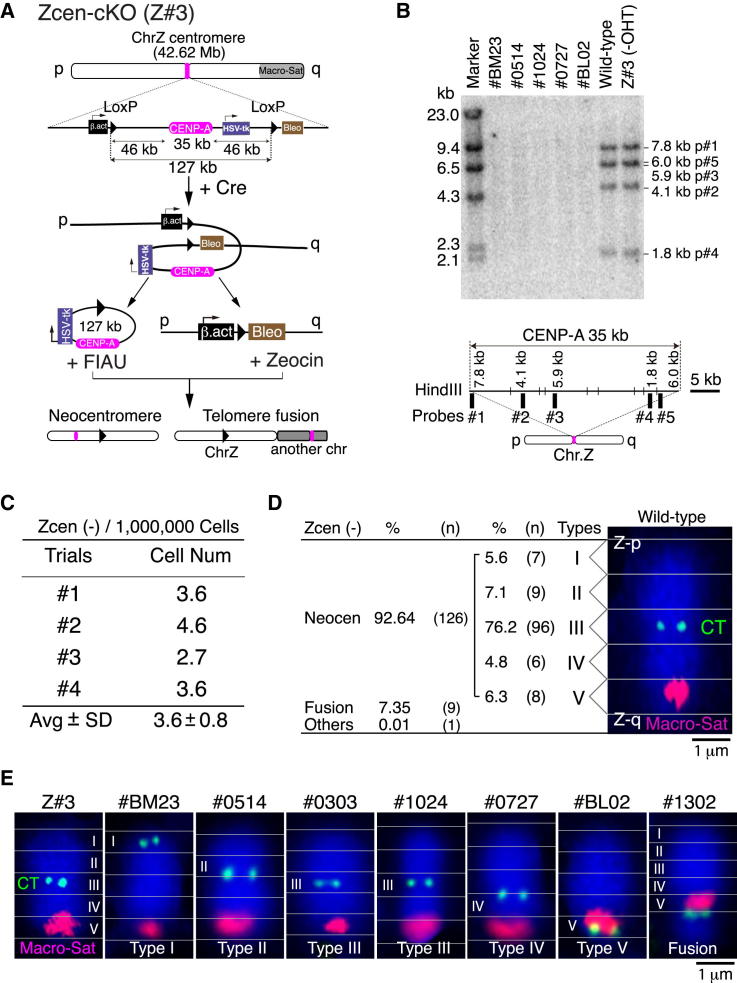
Formation of Neocentromeres on Chromosome Z (A) A strategy to isolate surviving cells after removal of endogenous centromere of chromosome Z with positive (Zeocin resistance) and negative (FIAU resistance) selections. Surviving cells are expected to have chromosome Z with a neocentromere or fusion chromosome Z with another chromosome. (B) Southern hybridization analysis to confirm that endogenous centromere sequence is removed. Probe information (probe #1–5) for Southern analysis to confirm removal of endogenous centromere is also shown. (C) Frequency of isolation of surviving cells without endogenous centromere sequence. We independently performed four trials. (D) Classification of surviving clones based on cytological experiments using anti-CENP-T (CT) antibodies as a centromere marker. Among 136 clones, ten clones have fusion chromosome Z with another chromosome. We classified the centromere position defined by CENP-T staining as five types (type I–V). (E) Immuno-FISH images of various types of chromosomes. Red shows signals from the satellite sequence on q-arm of chromosome Z. Centromeres (green) are visualized by anti-CENP-T antibodies. See also Figure S1.

To generate DT40 cell lines with neocentromeres, we conditionally removed a 127 kb region including the 35 kb CENP-A domain from the single chromosome Z (Figure 1A) and selected for cells that maintained this chromosome despite the loss of the centromere. Chromosome Z was chosen for this study because it is one of three chicken chromosomes whose CENP-A domain does not contain repetitive sequences. Also, because it is present as a single copy, the genomic data are not an amalgam of data from two alleles. Other chicken centromeres are assembled on DNA containing a variety of repetitive sequence elements (Shang et al., 2010). Despite differences in the underlying DNA sequence, the Z kinetochore appears to resemble those on other chromosomes with respect to the levels of a number of kinetochore proteins detected by quantitative immunofluorescence (Figure S1 available online).

To isolate neocentromeres, we first generated parental cell line Z#3 by inserting LoxP sequences flanking the Z centromere and expressing Mer-Cre-Mer recombinase (Gascoigne et al., 2011; Hori et al., 2013; Shang et al., 2010), which is activated by hydroxytamoxifen (OHT). As expected, most Z#3 cells died after OHT addition.

Next, we used a positive-negative double selection to isolate cells that retained the Z chromosome following excision of its endogenous centromere. For positive selection, we inserted a β-actin promoter sequence upstream of the 5′ LoxP site and a Bleomycin (Zeocin) resistance gene downstream of the 3′ LoxP (Figure 1A). For negative selection, we inserted the Herpes Simplex Virus thymidine kinase (*HSV-tk*) gene within the centromere near the 35 kb CENP-A-associated region. Cells expressing *HSV-tk* are sensitive to 5-iodo-2′-fluoroarauracil (FIAU). Thus, following excision of the endogenous centromere, Z#3 cells retaining chromosome Z are resistant to both Zeocin and to FIAU. Using this protocol, we isolated 136 surviving colonies after OHT addition (Figures 1 and S1), yielding a mean frequency of 3.6 × 10^−6^ (Figure 1C). Southern hybridization using multiple probes confirmed removal of the 127 kb region including the 35 kb endogenous centromere sequence in these surviving cells (Figures 1B, S1C, and S1D).

We used immunofluorescence combined with fluorescence in situ hybridization (Immuno-FISH) analysis with anti-CENP-T antibodies and a Z-specific macrosatellite probe (Hori et al., 1996) to confirm neocentromere formation and mapped the neocentromere positions in the surviving cells. The probe hybridizes to a large heterochromatic telomere-proximal region on the q-arm of chromosome Z (Macro-sat, red in Figure 1D), whose endogenous centromere is centrally located (CENP-T: CT, green in Figure 1D). We subdivided the length of chromosome Z in surviving cells into five equal regions, classifying the centromere position defined by CENP-T staining as p-telomere (type I), p-arm (type II), metacentric (type III), q-arm (type IV), and q-telomere (type V) (Figures 1D, 1E, and S2). We characterized all 136 surviving clones and found that 126 of them acquired neocentromeres, which formed in all regions of the chromosome (Figure 1D). Interestingly, 76% of neocentromeres were metacentric (type III), indicating a strong preference for neocentromere formation in this region of the chromosome. In a few clones (<10%), the acentric Z had fused with another chromosome and presumably segregated under control of that endogenous centromere (Figures 1D and S2).

### Functional Properties of Neocentromeres in the Surviving Cells

**Figure 2:**
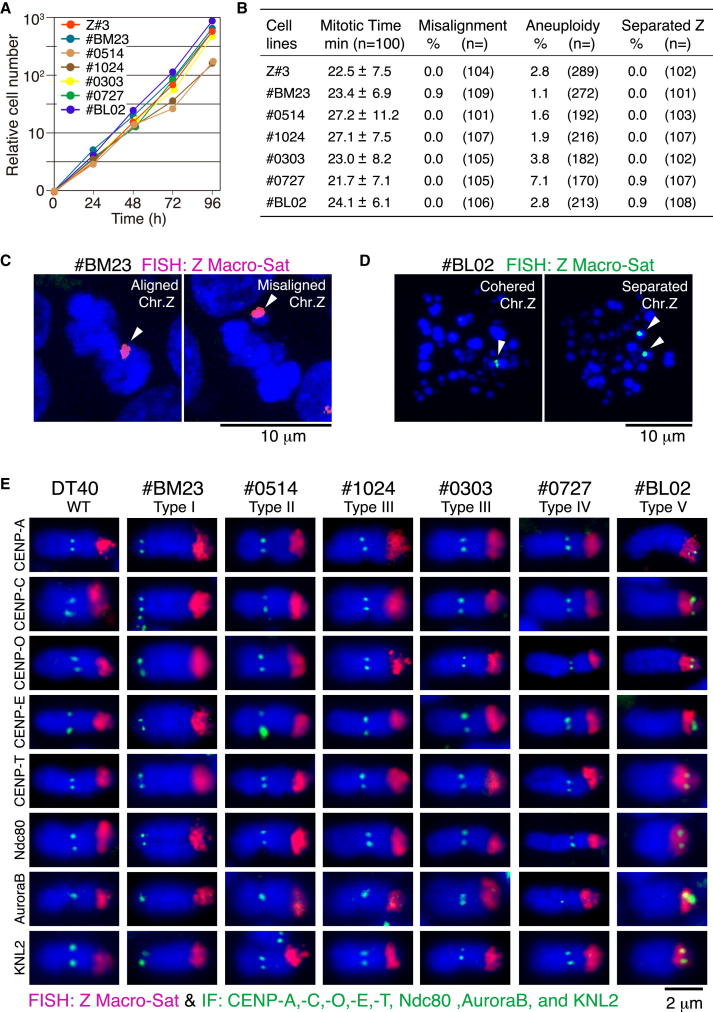
Cells with Neocentromere Show Normal Chromosome Segregation (A) Growth curve of cells with neocentromere. Z#3 has endogenous centromere on chromosome Z (control). #BM23, #0303, #0727, #BL02, #0514, and #1024 cells, each of which has a neocentromere on chromosome Z, grew well as Z#3 cells. (B) Time to complete mitotic progression, population of cells with misaligned chromosome, percentage of aneuploid cells, and numbers of cells in which sister chromatids of chromosome Z are prematurely separated in each neocentromere-containing cell line. (C) Typical images of cells with either aligned or misaligned chromosome Z. Chromosome Z was detected by FISH the satellite sequence on q-arm of chromosome Z as a probe (red). (D) A typical image of cell in which sister chromatids of chromosome Z are prematurely separated during prometaphase (right). Left shows a cohered chromosome Z (control). (E) Immunofluorescence analysis on neocentromeres with antibodies against CENP-A, -C, -O, -E, -T, Ndc80, Aurora B, and KNL2. All tested centromere proteins were detected on all neocentromeres. See also Figure S2.

Most cell lines with neocentromeres grew at a similar rate to wild-type DT40 cells (Figure 2A), suggesting that the newly formed neocentromeres were functionally equivalent to endogenous centromeres. We measured how long cell lines with neocentromeres took for mitotic progression by live-cell imaging and found that their mitotic progression appeared normal, as these cells went through mitosis with a timing similar to wild-type DT40 cells (Figure 2B). FISH analysis revealed that there was no significant decrease in the stability of the neocentromere-containing chromosome Z (Figure 2B).

To further test the functionality of neocentromeres, we identified cells with metaphase plates and scored the number of cells having a misaligned chromosome Z. This analysis revealed no increase in numbers of misaligned chromosome Z in neocentromere-containing cells (Figures 2B and 2C). Consistent with this, immunostaining of six neocentromeres with antibodies against multiple kinetochore proteins and the inner centromere protein Aurora B revealed that these neocentromeres apparently assemble a functional kinetochore structure similar to endogenous centromeres (Figure 2E).

Some human neocentromeres exhibit weakened sister chromatid cohesion, giving rise to premature sister chromatid separation following extended incubation with nocodazole (Alonso et al., 2010; Bassett et al., 2010). However, we did not detect premature separation of chicken neocentromeres following incubation of cells with nocodazole for 14 hr (Figures 2B and 2D).

Together, these functional characterization experiments suggest that neocentromeres in DT40 cells are functionally equivalent to endogenous centromeres. However, we cannot completely rule out subtle defects in neocentromeres that are not detected by our experiments.

### Neocentromeres Assemble CENP-A on ∼40 kb Regions

**Figure 3:**
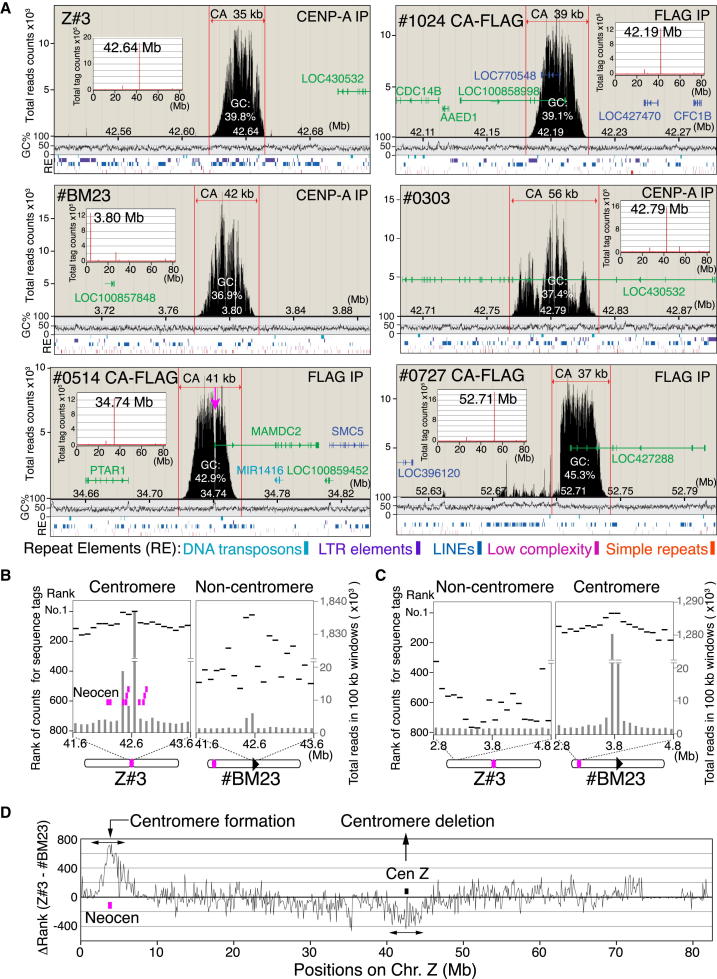
Neocentromeres Are ∼40 kb Long (A) ChIP-seq analysis with anti-FLAG or anti-CENP-A antibodies in cells containing neocentromere. Size of the CENP-A domain in each cell line was shown. We used anti-FLAG antibodies for cells expressing CENP-A-FLAG (FLAG-IP). For cells not expressing CENP-A-FLAG, we used native CENP-A antibodies (CENP-A-IP). IP DNAs were deeply sequenced and sequence data were mapped on chicken genome database. We first identified a major peak as a neocentromere (each position is indicated) from entire chromosome Z in each cell line and examined detail distribution around the peak with a higher resolution. GC% contents and distribution of transposons, repeat sequence, and genes in CENP-A-associated DNAs are shown. Arrow in data of #0514 cells indicates a gap of CENP-A distribution, which corresponds to exon 1 of *MAMDC2* gene (also see Figure 7). (B) Counts of sequence reads (gray bar) for CENP-A-IP DNAs around centromere (Z#3) and noncentromere (#BM23) region. Ranking for these counts are also shown (black line). Pink dots are shown as neocentromere loci. CENP-A-associated DNAs were enriched in 2 Mb around the major CENP-A peak (Z#3). However, numbers of sequence tags associated with CENP-A were reduced in this region, when an endogenous centromere was removed (#BM23). (C) Counts of sequence reads (gray bar) for CENP-A-IP and their ranking (black line) around centromere (#BM23) and noncentromere (Z#3) region. CENP-A cluster was observed around centromere (#BM23). (D) Genome-wide ranking of numbers of sequence tags associated with CENP-A for Z#3 cells were subtracted from that for #BM23. A CENP-A cluster was observed around neocentromere region of #BM23. See also Figures S3 and S4.

Following cytogenetic mapping of neocentromere positions (Figure 1), we used ChIP-seq analysis to analyze CENP-A-associated DNAs and map the neocentromere locations with higher precision. ChIP-seq analysis previously indicated that the endogenous CENP-A-associated region spans a 35 kb region on chicken chromosome Z (Shang et al., 2010). We performed ChIP-seq analysis with endogenous anti-CENP-A or anti-FLAG antibodies for 18 neocentromere cell lines and found in each case a single clear CENP-A-associated region similar in size to the endogenous Z centromere (Z#3). While CENP-A peak positions varied among neocentromere-containing cell lines, peak sizes were remarkably similar with an average of 41 ± 5.9 kb (Figures 3 and S3A).

We were concerned that the size of the CENP-A domain might potentially be influenced by the overexpression of exogenous CENP-A-FLAG. However, when we compared the CENP-A distribution of cells expressing CENP-A-FLAG with that of cells expressing only endogenous CENP-A, we found that the CENP-A distribution is similar even in cell lines in which the CENP-A-FLAG is slightly overexpressed (Figures S3B and S3C). We conclude that ∼40 kb is sufficient to establish a structural foundation for kinetochore assembly in chicken cells.

Marshall et al. (2008) previously proposed that human neocentromeres preferentially form on AT-rich sequences. We therefore determined the GC% content of the 18 neocentromeres mapped in detail. The overall GC% content of the entire chicken Z chromosome (∼81 Mb) is 41%. The GC% content of 14/18 mapped neocentromeres was typically less than 41% (Figures 3A and S3). Thus, chicken neocentromeres may form preferentially on sequences with higher than average AT% content.

Human neocentromeres are enriched in LINE elements (Chueh et al., 2009). Although some chicken neocentromeres (e.g., #1109; Figure S3A) and the native centromere Z (Z#3; Figure 3) are LINE rich, others (e.g., #2418 or #1320; Figure S3A) are LINE poor. In addition, we observed no enrichment of DNA transposons in the 18 tested neocentromeres. Overall, we observed no bias toward any known repetitive DNA elements in neocentromeres. Harr-plot analysis to search for sequences common among the 18 mapped neocentromeres failed to reveal any significant local sequence homologies (data not shown). From these analyses, we conclude that neocentromeres do not exhibit any detectable DNA sequence preference.

### The CENP-O or CENP-S-X Complexes Are Not Required for Efficient Neocentromere Formation

Many centromere-associated proteins are essential for cell growth and kinetochore formation, and cells with knockouts of those proteins are not viable due to strong mitotic defects (Perpelescu and Fukagawa, 2011). However, DT40 cells depleted for CENP-O complex proteins or CENP-S-X proteins are viable, although certain aspects of kinetochore function are compromised (Amano et al., 2009; Hori et al., 2008b; Perpelescu and Fukagawa, 2011). To examine the contribution of CENP-O and CENP-S-X complex proteins to neocentromere formation, we performed the neocentromere assay in cells deficient for CENP-P or CENP-S by knocking out the relevant genes in Z#3 cells. We confirmed that the target proteins are depleted in these knockout cells (Figure S4A) and then performed the neocentromere assay. Remarkably, the frequency of neocentromere formation in CENP-P- or CENP-S-deficient cells was similar to that in Z#3 cells (Figure S4B). Thus, the CENP-O and CENP-S-X complexes do not have essential roles in de novo centromere formation.

### CENP-A Incorporation at Low Levels in Chromatin Regions Flanking Endogenous Centromeres

Although neocentromeres can apparently form in any region of chromosome Z, 76% of them formed in regions adjacent to the excised original centromere region (Figure 1D). A tendency of neocentromeres to form immediately adjacent to the site of the excised native centromere was also observed following centromere deletion in the yeast *C. albicans* (Ketel et al., 2009). These observations, together with the lack of particular DNA sequences associated with neocentromeres, suggest that epigenetic marks flanking the original centromere might influence neocentromere formation. For example, the levels of CENP-A could be higher in regions flanking the original centromere relative to other regions of the chromosome. To test this, we examined CENP-A levels across the central region of chromosome Z in endogenous centromere (Z#3) or neocentromere-containing (#BM23) cell lines by ChIP-seq and scoring the number of CENP-A-associated sequence tags.

As shown in Figure 3B, a 2 Mb region surrounding the Z centromere region (position 42.6 Mb of ChrZ in Z#3) displayed a consistently high number of CENP-A-associated sequence tags identified by this method. However, this enriched cluster in the vicinity of position 42.6 Mb was not detected in #BM23 cells, in which the 127 kb flanking the original centromere had been removed and a neocentromere had formed at a different locus (Figure 3B). Instead, in #BM23 cells a CENP-A-enriched cluster was detected around the neocentromere, at a site where CENP-A levels were low in parental Z#3 cells (Figure 3C). We also compared the number of CENP-A-associated sequence tags of #BM23 cells with that of Z#3 by a genome-wide difference analysis (Figure 3D) and confirmed that a CENP-A-enriched region formed around the neocentromere, while levels of CENP-A enrichment fell near the original centromere.

These data demonstrate that a consistently high number of CENP-A-associated sequence tags are detected flanking centromeres independent of the underlying primary DNA sequence, suggesting that low levels of CENP-A may spread to regions adjacent to established centromeres. These levels of CENP-A may normally be too low to nucleate kinetochore structure. However, if the region with the highest CENP-A concentration (the endogenous centromere) is removed, one or more of the latent CENP-A “seeds” in the flanking region might become established and direct neocentromere formation.

**Figure 4:**
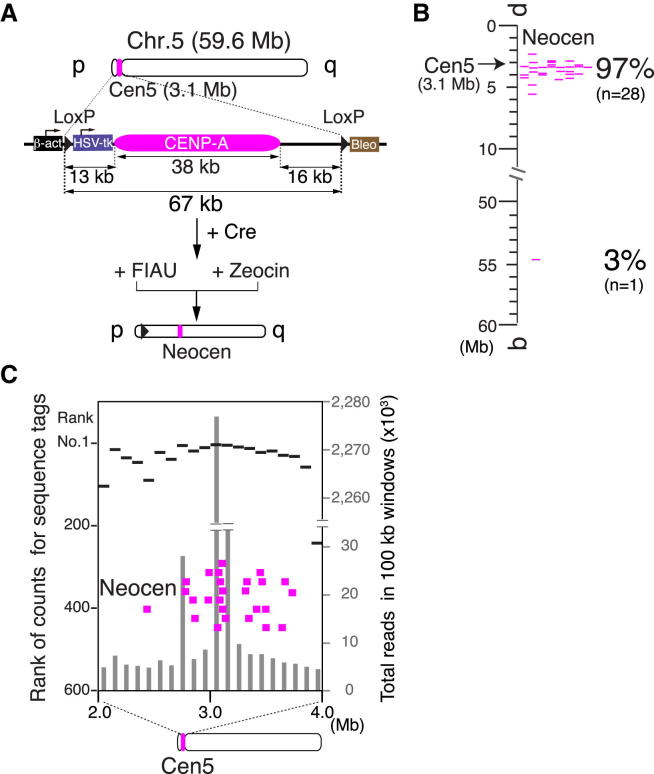
Neocentromere Formation on Chromosome 5 (A) A strategy to isolate surviving cells after removal of endogenous centromere of chromosome 5 with positive (Zeocin resistance) and negative (FIAU resistance) selections. In this case, we used shorter deletion (67 kb) than for Z centromere deletion (127 kb). (B) Location of neocentromeres on chromosome 5. These locations were determined by ChIP-seq analysis with anti-CENP-A. Ninety-seven percent of neocentromeres are located in the 3 Mb region from the original centromere. (C) Counts of sequence reads (gray bar) for CENP-A-IP and their ranking (black line) around centromere 5. A CENP-A cluster was observed around centromere and neocentromeres (pink) are formed in the CENP-A cluster region.

To further test this hypothesis, we developed a strategy to excise a smaller region encompassing the centromere of chromosome 5 (Cen5) and isolate neocentromere-containing cells. In this experiment, we deleted 67 kb including the CENP-A domain, a region about half the size of the 127 kb that we removed from CenZ (Figure 4A). If our hypothesis were correct, the 67 kb deletion should leave behind much more CENP-A-enriched chromatin near the deleted CENP-A domain and neocentromeres should form even more efficiently near the original centromere. In this experiment, we analyzed 29 neocentromeres by ChIP-seq with anti-CENP-A and found that 28 (97%) of them formed within a 3 Mb region surrounding the original centromere 5 (Figures 4B and 4C).

In summary, we conclude that nonkinetochore CENP-A is normally scattered flanking the core kinetochore sequence. This CENP-A, which has been mapped here, can apparently function to seed neocentromere formation if the original dominant centromeric CENP-A domain is removed or lost.

### Chromatin Landscape around Neocentromeres

In many organisms, native centromeres are composed of CENP-A-rich kinetochore-associated centromeric chromatin flanked by heterochromatin (Allshire and Karpen, 2008). In *S. pombe* or *Drosophila* cells, heterochromatin facilitates kinetochore formation (Folco et al., 2008; Ishii et al., 2008; Olszak et al., 2011). In contrast, the heterochromatin mark histone H3 trimethylated on K9 (H3K9me3) is not enriched around human neocentromeres (Alonso et al., 2010). In addition, de novo centromere formation occurs independently of heterochromatin formation in *C. elegans* (Yuen et al., 2011).

**Figure 5:**
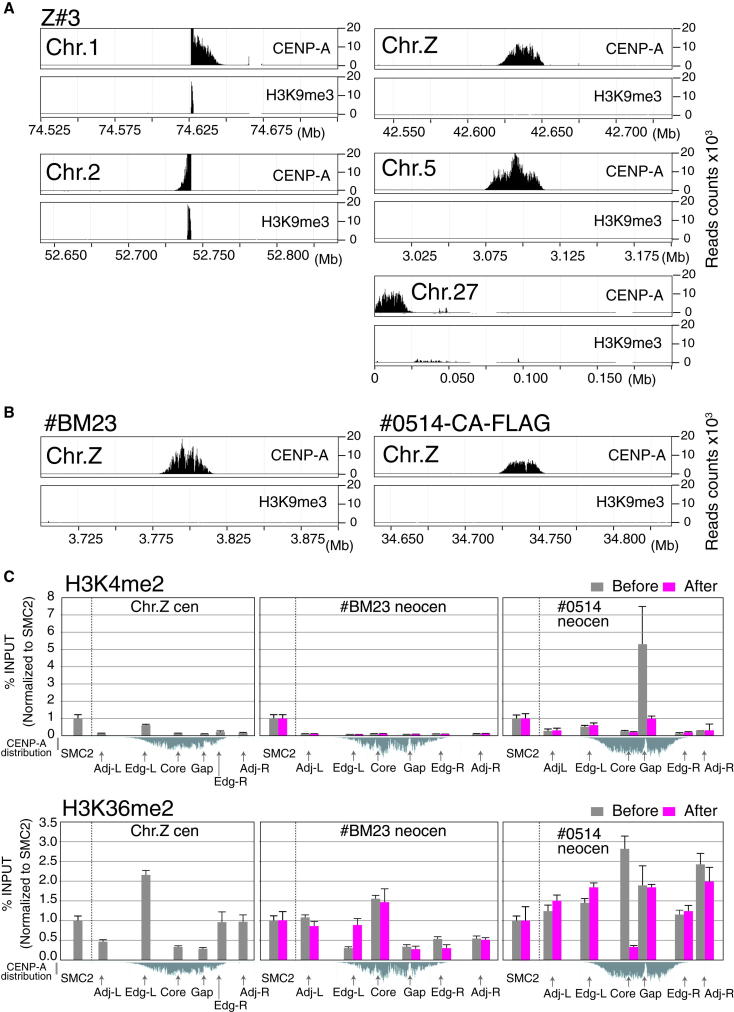
Chromatin Features in Various Neocentromeres (A) ChIP-seq analysis with anti-H3K9me3 or anti-CENP-A in Z#3 cells. In chromosomes 1 and 2 that have repetitive centromeres, H3K9me3 was enriched around the CENP-A domain. H3K9me3 was not enriched around nonrepetitive centromeres of chromosomes Z, 5, and 27. (B) ChIP-seq analysis with anti-H3K9me3 or anti-CENP-A in neocentromere-containing #BM23 and #0514 cells. H3K9me3 was not enriched around neocentromere region in both cell lines. (C) ChIP-PCR analysis with antibodies against H3K4me2 and H3K36me2 around centromere regions in Z#3 (chromosome Z), #BM23 (Z neocentromere), and #0514 (Z neocentromere). In #BM23 and #0514 cells, these profiles are shown before and after neocentromere formation. Primer positions for this analysis and ChIP-seq data with anti-CENP-A antibodies in each cell line are also shown. Error bars show SEM. See also Figure S5.

We performed ChIP-seq analysis with anti-H3K9me3 to examine the heterochromatin state of chicken centromeres, which are based either on (most commonly) repetitive or nonrepetitive DNA sequences (Shang et al., 2010). We detected substantial levels of H3K9me3 at the repetitive centromeres of chromosomes 1 and 2 but found no significant accumulation of H3K9me3 around the nonrepetitive centromeres of chromosomes 5, 27, and Z (Figure 5A). This analysis was confirmed by indirect immunofluorescence, in which we detected substantial levels of H3K9me3 at native centromeres with repetitive sequences including Cen1, 2, 3, and 4 and over the entirety of chromosome W, which is highly enriched in repetitive sequences (Hori et al., 2000). In contrast, we did not detect strong H3K9me3 signals at the nonrepetitive Z centromere (Figure S5).

We next investigated the distribution of H3K9me3 around neocentromeres in cell lines #BM23 and #0514. As was the case for the nonrepetitive centromeres 5, 27, and Z, we did not detect significant accumulation of H3K9me3 around the CENP-A domain (Figure 5B). Heterochromatin is thought to facilitate recruitment of the chromosome passenger complex (CPC). Despite the absence of clearly enriched H3K9me3, we readily observed CPC localization at neocentromeres (Figure 2E). We speculate that repetitive sequences may promote heterochromatin formation, but that neither repetitive sequences nor heterochromatin is essential for kinetochore formation and function in vertebrates.

A unique aspect of our experimental system is that it allows us to examine the chromatin organization of chromosomal loci before and after those loci become neocentromeres in the same cell line. To analyze the chromatin state of nonrepetitive centromeres during neocentromere formation, we performed ChIP-PCR analysis with antibodies against H3K4me2 and H3K36me3 in #BM23 (neocentromere) or #0514 (neocentromere) and Z#3 (parental) cell lines. H3K4me2 and H3K36me3 have been suggested to be important components of centromeric chromatin (Bergmann et al., 2011; Sullivan and Karpen, 2004). As a control, we characterized the chromatin state of the housekeeping gene for condensin subunit SMC2 (also located on chromosome Z). We found no centromere-specific accumulation of H3K4me2 or H3K36me2 at any of the centromeres (Figure 5C), suggesting that these are not universal obligate markers of centromere chromatin and that the histone modification status of kinetochore-associated chromatin is plastic.

In one neocentromere cell line (#0514), the neocentromere covers the 5′ end of the *MCMDC2* gene, which is expressed in DT40 cells. In precursor Z#3 (wild-type) cells, we detected high levels of H3K4me2 and H3K36me2 over this gene. Both histone marks were strongly reduced upon neocentromere formation (#0514 cells, Figure 5C). This reduction in H3K4me2 and H3K36me2 levels upon neocentromere #0514 formation suggests that transcription levels may be reduced upon neocentromere formation (discussed in detail in the last section).

### DNA Replication Timing for Neocentromere Regions

It has been suggested that centromere formation may be linked to the specific patterns of DNA replication of chromosomal domains (Ahmad and Henikoff, 2001; Shelby et al., 2000; Sullivan and Karpen, 2001). Experimentally generated neocentromeres can provide important insights into possible links between centromere formation and DNA replication timing, because we can test the DNA replication timing of a locus before and after neocentromere formation. Furthermore, because neocentromeres lack highly repeated DNA sequences, we can use DNA microarrays to measure DNA replication timing at high resolution.

**Figure 6:**
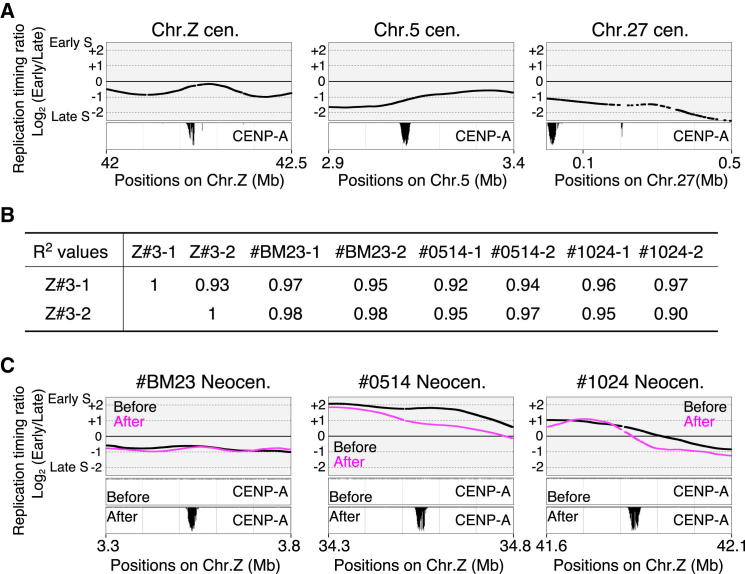
DNA Reapplication Timing of Neocentromeres (A) DNA replication profiles around endogenous centromere regions on chromosomes Z, 5, and 27 in DT40 cells. Endogenous centromeres are on middle to late replication domain. (B) Comparison of data of entire DNA replication timing for Z#3 (before neocentromere formation) with those for BM23, #0514, and #1024 cells (after neocentromere formation). *R*^2^ values are shown. (C) Changes of DNA replication timing at three neocentromere loci before and after neocentromere formation. By SAM, later shifts in replication timing of 100 kb segments at loci of neocentromere formation in both #0514 and #1024 cells were found to be statistically significant (#0514: p = 1.86 x 10^−5^, q = 0.0153; #1024: p = 7.79 x 10^−3^, q = 0.0903; q value is a FDR-based measure of significance; Storey and Tibshirani, 2003). Replication timing was not changed in #BM23 cells upon neocentromere formation.

We pulse-labeled neocentromere-containing DT40 cells with bromodeoxyuridine (BrdU) and separated cells into early and late S phase fractions by flow cytometry. Next, BrdU-substituted DNA from each fraction was recovered by immunoprecipitation with an anti-BrdU antibody, differentially labeled, and cohybridized to a chicken whole-genome oligonucleotide microarray. This analysis revealed that native centromeres (Cen5, 27, Z) generally replicated during the latter half of S phase (Figure 6A). This is in contrast to data obtained in yeasts, in which centromere regions replicate early in S (Koren et al., 2010; Raghuraman et al., 2001), but is consistent with results in mammals where centromere-associated alpha-satellite DNA and a neocentromere were both shown to replicate in mid/late S (Lo et al., 2001; O’Keefe et al., 1992).

We next examined replication timing at three loci before and after neocentromere formation. Genome-wide replication profiles are remarkably similar before and after neocentromere formation (see Figure 6B; *R*^2^ values ranged between 0.90 and 0.98 after loess smoothing). Indeed, in #BM23 cells, neocentromere formation in a late replicating domain of the Z chromosome did not alter the replication timing of that domain (Figure 6C). However, in #0514 or #1024 cells, neocentromeres formed on domains of the Z chromosome that replicated in early or mid-early S, respectively. Both domains shifted to a later replication timing after neocentromere formation (Figure 6C). We confirmed the significance of these changes using significance analysis of microarrays (SAMs) (Tusher et al., 2001) (see Experimental Procedures and Supplemental Information).

In *C. albicans*, a change of DNA replication timing from late to early was observed upon neocentromere formation. This was interpreted as suggesting that centromere formation facilitated binding of the origin recognition complex (ORC) to centromere loci (Koren et al., 2010). However, considering the DNA replication timing of native centromeres and three neocentromere loci measured here, we hypothesize that centromere formation does not facilitate early DNA replication in vertebrate cells.

### Neocentromeres Form on Both Transcriptionally Active and Inactive Chromosome Loci

**Figure 7:**
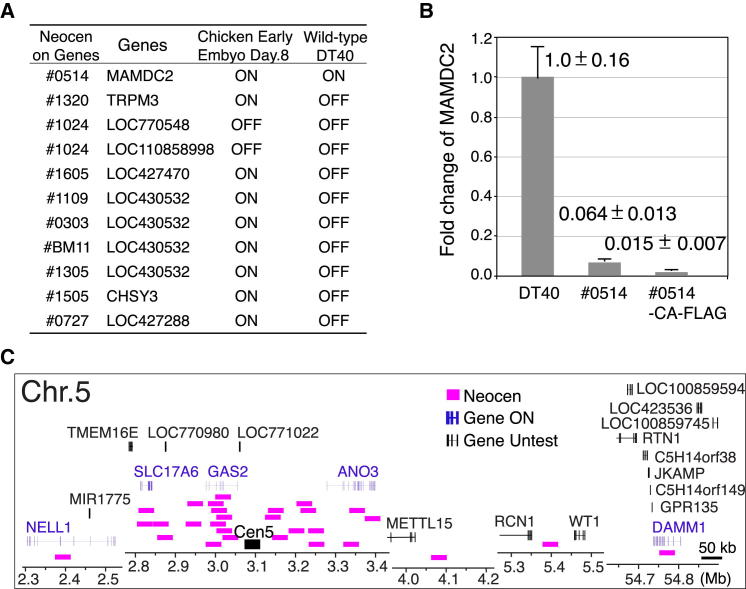
Neocentromeres Are Efficiently Formed on Nontranscribed Region in Chromosome Z (A) Examination of expression for genes, which are located on each neocentromere, in early embryos or wild-type DT40 cells. Expression was analyzed by RT-PCR. Most genes except for *MAMDC2* are not expressed in DT40 cells, but expressed in early embryonic cells. (B) Quantitative-RT-PCR analysis of *MAMDC2* in wild-type DT40 or #0514 cells in which a neocentromere is formed on *MAMDC2* gene locus (see Figure 3A). #0514-CAf cells are expressing CENP-A-FLAG. Expression level of *MAMDC2* extremely was reduced in #0514 cells. Error bars show SD. (C) Position of each neocentromere and native centromere on chromosome 5. Location of genes around neocentromeres is shown. Many neocentromeres are formed on transcribed genes in chromosome 5.

ChIP-seq analysis with CENP-A for our library of neocentromeres revealed that neocentromeres could form on gene-coding regions (Figures 3A and S3). Indeed, 10 of the 18 tested neocentromeres formed on eight distinct genes on the Z chromosome (some neocentromeres overlapped; see Figure 7A). To characterize the relationship between gene expression and neocentromere formation, we analyzed the expression levels of these eight genes in wild-type DT40 and early embryonic cells by RT-PCR. Remarkably, although six transcripts from these genes were detected in early embryonic RNA, only the *MAMDC2* gene was expressed in DT40 cells. Two genes were not expressed in either cell type (Figure 7A). As approximately 62% of total genes are expressed in DT40 cells (Neiman et al., 2006), this appeared to suggest that neocentromeres preferentially form on nontranscribed regions in Z chromosome.

This apparent preference for neocentromere formation on nontranscribed chromatin could arise as a consequence of there being only a single Z chromosome in DT40 cells. If centromere formation suppressed transcription of a gene within the centromere locus and this gene were essential for cell viability, neocentromere formation at that site might knock down gene expression and result in cell death. Thus, our assay would preferentially select cell lines in which neocentromeres formed in nontranscribed or gene-free regions.

Detailed analysis of the ChIP-seq profile of CENP-A in #0514 cells revealed a gap in the CENP-A peak (arrow in Figure 3A). This corresponded to exon 1 of the *MAMDC2* gene and suggested that there could be a conflict between centromeric chromatin and high levels of transcription. Indeed, *MAMDC2* expression was reduced 20- to 100-fold relative to wild-type DT40 cells in #0514 cells in which the 5′ end of the gene was covered by the CENP-A domain of the neocentromere (Figure 7B). Thus, at least in this instance, gene expression was strongly suppressed upon neocentromere formation.

To further test the hypothesis that neocentromeres can form at both active and inactive chromosomal loci, we characterized the positions of neocentromeres on chromosome 5 (Figure 4). As DT40 is diploid, cells have two copies of chromosome 5. There should thus be no selection against neocentromere formation on actively transcribed essential genes. Remarkably, we found that 15/29 neocentromeres formed on chromosome 5 within regions of transcribed genes (Figure 7C). Unfortunately, because the dearth of genomic resources for chicken (e.g., high-resolution SNP maps) prevents us from distinguishing between the two alleles of these genes, we have not been able to determine whether transcript levels also drop for these genes.

The results of these studies reveal that active chromatin is not a barrier to neocentromere formation, but support previous studies showing that centromeric chromatin and efficient transcription are mutually incompatible (Bergmann et al., 2012).

## Discussion

Here, we report a chromosome-engineering approach to create neocentromeres in chicken DT40 cells. This approach has allowed us to explore several key questions, including the relationship between transcription and centromere/kinetochore formation, the ability of centromeres to alter the replication timing of chromosomal domains, and the role of noncentromeric CENP-A in nucleating neocentromere formation.

### Where and How Do Neocentromeres Form?

Neocentromere formation has been observed in many organisms including plants, fungi, insects, and vertebrate cells. Comparison of neocentromeres in diverse organisms reveals some features that are common among species, but also other organism-specific features. In plants, “rescue” chromosomes isolated following centromere inactivation by chromosome rearrangements form neocentromeres at multiple positions on chromosome arms (Nasuda et al., 2005; Topp et al., 2009). Human neocentromeres are also located at multiple positions (Marshall et al., 2008). In contrast, most neocentromeres form near the original centromere in both *C. albicans* (Ketel et al., 2009) and *D. melanogaster* (Maggert and Karpen, 2001).

We observed neocentromere formation at multiple positions in chicken cells, but most formed close to original centromeres. When we deleted 127 kb encompassing the Z centromere, 76% of neocentromeres formed in the region close to the deletion site (Figure 1). When we used a shorter deletion (67 kb) to remove centromere 5, 97% of neocentromeres formed in the 3 Mb flanking the deletion site based on ChIP-seq analysis. These results suggest that epigenetic/chromatin marks favoring neocentromere formation are clustered near the original centromere.

Considering our results together with observations in other systems, we propose that neocentromeres can form anywhere along the chromosome, but because potential epigenetic marks are enriched around the original centromeres, neocentromeres exhibit a preference for formation in that region. Our data strongly suggest that one such epigenetic/chromatin mark could be nonkinetochore CENP-A incorporated around the original centromeres, which we have here been able to map because the nonrepetitive underlying sequences make high-resolution mapping possible (Figure 3). Discovery of this nonkinetochore CENP-A, and the evidence suggesting that it can nucleate neocentromere formation, raises a very interesting issue for future exploration, namely, the mechanism by which centromere spreading or the nucleation of ectopic centromeres is prevented in wild-type cells. As we will discuss below, chickens exhibit a remarkably narrow range of kinetochore sizes, at least on centromeres assembled on nonrepetitive DNA.

As is the case for human and *C. albicans* neocentromeres, chicken neocentromeres are not associated with heterochromatin. This contrasts with the situation in *D. melanogaster* and *S. pombe*, where heterochromatin facilitates neocentromere formation (Folco et al., 2008; Ishii et al., 2008; Olszak et al., 2011). Heterochromatin is highly enriched in pericentromeric regions around all human natural centromeres (Lam et al., 2006). In chicken, most centromeres assemble on complex repeated DNA sequences that are flanked by heterochromatin containing H3K9me3. However, this histone mark does not accumulate around nonrepetitive native centromeres in chicken or neocentromeres in chicken and human (Alonso et al., 2010).

Although heterochromatin flanking kinetochore regions is thought to facilitate sister-chromatid cohesion and recruitment of the CPC, both we and Bassett et al. (2010) observed correct CPC localization at neocentromeres. We speculate that repetitive sequences may promote heterochromatin formation, but our data together with other studies of neocentromeres suggest that neither repetitive sequences nor heterochromatin is essential for kinetochore formation or function in vertebrates and *C. albicans* (Alonso et al., 2010; Ketel et al., 2009; Marshall et al., 2008).

Once neocentromeres form at a normally euchromatic locus, the resulting centromeric chromatin is maintained through multiple cell cycles and rounds of chromosome segregation. This stability requires an epigenetic marker such as CENP-A, but it has come to be believed that other chromatin marks such as H3K4me2 and H3K36me3 are important constituents of centromeric chromatin in human and *D. melanogaster* neocentromeres (Bergmann et al., 2011; Sullivan and Karpen, 2004). However, H3K4me2 accumulation was not observed in plant centromeres (Jin et al., 2008). Here, we did not detect accumulation of H3K4me2 and H3K36me3 at chicken natural centromeres or neocentromeres. This suggests that centromere chromatin may be more plastic than previously assumed.

### Remarkable Conservation of Neocentromere Size

The size of the centromeric CENP-A domain varies widely in human cells. For example, Sullivan et al. (2011) found that the CENP-A domain on endogenous human alpha satellite arrays ranged from 180 kb to 2 Mb. The smallest values measured for native human centromeres resembled those found for human neocentromeres (Alonso et al., 2007). In addition, Lam et al. (2006) suggested that CENP-A-containing chromatin can expand across Mb regions at highly repetitive centromeres. Neocentromeres of *C. albicans* also vary in size (Ketel et al., 2009).

Our ChIP-seq analysis of 18 chicken neocentromeres revealed that the CENP-A-associated region for all of them was quite constant: 41 ± 5.9 kb long. This is in remarkable agreement with our previous observation that the CENP-A domain of natural centromeres of chicken chromosomes Z, 5, and 27 is 30–40 kb (Shang et al., 2010). Considering these results, we conclude that the functional size of chicken unique sequence centromeres is a remarkably conserved 30–40 kb. These observations raise the intriguing possibility that chicken cells have a mechanism to keep centromere size constant. This suppression by an established centromere of the seeding of neocentromeres elsewhere on the chromosome may be analogous to the phenomenon of recombination interference in meiotic chromosomes. Determination of the as-yet-unknown mechanism is an important subject for future studies.

### Neocentromere Formation Is Not Associated with Early DNA Replication in Vertebrate Cells

Fungal centromeres including *S. cerevisiae*, *S. pombe*, and *C. albicans* undergo DNA replication early in S phase. Furthermore, in *C. albicans* the replication timing of a chromosome domain shifts to earlier in S phase upon neocentromere formation (Koren et al., 2010). As a result of the yeast studies, it has been proposed that early DNA replication may be coupled with CENP-A incorporation (Koren et al., 2010). However, as CENP-A incorporation occurs in early G1 in both human and chicken cells (Silva et al., 2012), it is not obvious how early DNA replication would be coupled to CENP-A deposition in vertebrate cells.

The replication timing for vertebrate centromeres appears to differ from that in yeasts. For example, alpha-satellite DNA in human cells replicates in mid-S phase (O’Keefe et al., 1992), as did the CENP-A domain of a neocentromere (Lo et al., 2001). Interestingly, in the second study, neocentromere formation caused sequences adjacent to the CENP-A domain to shift to a later replication timing.

Our studies reveal that chicken centromeres replicate relatively late in S phase. Furthermore, the replication timing of a late replicating domain was not significantly changed when a neocentromere formed within it. In contrast, an early replicating domain shifted to a later replication timing following neocentromere formation within it. The mechanism by which neocentromere formation influences the replication timing of the associated chromatin domain remains to be identified. These neocentromere cell lines thus offer a good system for analysis of the regulation of replication timing.

### Neocentromeres Can Be Formed within Transcriptionally Active Region

Until recently, accepted dogma has been that centromeres are nontranscribed heterochromatic regions. Indeed, this view is still held for *C. elegans* germ cells, where centromere chromatin forms efficiently on nontranscribed regions, which serve as marks for centromere positioning (Gassmann et al., 2012), and in *S. pombe*, where heterochromatin was shown to be necessary for de novo centromere formation (Folco et al., 2008). This view of centromeres as transcriptionally quiescent began to be challenged when active transcription was detected in plant centromeres (Nagaki et al., 2004) and noncoding RNA was found to be essential for heterochromatin formation near centromeres (Fukagawa et al., 2004; Volpe et al., 2002). Subsequently, marks characteristic of transcribed chromatin were found in human centromeric chromatin (Bergmann et al., 2011; Sullivan and Karpen, 2004), and, indeed, it was shown that seeding of heterochromatin could efficiently inactivate a human centromere (Cardinale et al., 2009; Nakano et al., 2008). More recently, it was suggested that expression of LINE RNAs may occur during human neocentromere formation (Chueh et al., 2009), and active polymerase II transcription has been detected in human centromeres during mitosis (Chan et al., 2012).

One important question that we could address using our chromosome-engineering assay was whether, as in *S. pombe*, inactive chromatin is necessary for de novo centromere formation. We find that it is not. Indeed, we could observe neocentromere formation on one actively transcribed locus of the Z chromosome, and 15 of 29 neocentromeres formed on chromosome 5 incorporated actively transcribed loci. In addition, the heterochromatin mark H3K9me3 was not associated with neocentromeres. This is not to say, however, that centromere formation is compatible with housekeeping levels of transcription. Indeed, for the *MAMDC2* locus on Z chromosome where transcript level can be quantitated, neocentromere formation was accompanied by a substantial suppression of transcription.

Our chromosome engineering system has allowed us to efficiently create neocentromeres in vertebrate cells. Despite extensive clinical studies, human neocentromeres have been reported in only 90 cases (Kalitsis and Choo, 2012), so this single study has more than doubled the number of vertebrate neocentromeres available for analysis. This experimental system provides a powerful resource for studies of centromere assembly, structure, and regulation and gives us valuable insights to understand how and where neocentromeres form.

## Experimental Procedures

### DT40 Culture and Immunoprecipitation of Neocentromere DNA

All DT40 cells were cultured at 38°C in Dulbecco’s modified medium supplemented with 10% fetal calf serum, 1% chicken serum, 2-mercaptoethanol, penicillin, and streptomycin (Hori et al., 2008a). Plasmid constructs were transfected with a Gene Pulser II electroporator (Bio-Rad) into DT40 cells. Detailed strategy for isolation of cells with neocentromere is shown in Figure S1. CENP-S- or CENP-P-deficient cells were created as described previously (Amano et al., 2009; Okada et al., 2006). We characterized 18 neocentromere-containing cell lines more detail. A FLAG-CENP-A construct was integrated into all cell lines.

### Functional Assay for Neocentromeres

To assess functional properties of neocentromeres, we examined time to complete mitosis, ability of chromosome alignment, chromosome stabilities, and sister chromatids separation for cells with neocentromeres. Time to complete mitosis was measured by live-cell imaging. Cells were stained with Hoechst 33342 for 10–15 min at a final concentration of 100 ng/ml, and time-lapse images of living cells were recorded at 5 min intervals with an exposure time of 0.2 s using a Confocal Scanner Box, Cell Voyager CV1000 (Yokogawa, Japan) with an oil immersion objective lens (PlanApo 60× objective lens, NA = 1.40). To observe ability of chromosome alignment, cells were treated with MG132 and counted with misaligned chromosome Z. Chromosome Z was detected by FISH. To see sister chromatids separation, metaphase spreads were prepared by methanol-acetic acid method after treatment of cells with nocodazole for 14 hr. Chromosome Z was detected by FISH using Z-specific satellite marker (Hori et al., 1996) as a probe.

### ChIP-Seq Analysis

For chromatin immunoprecipitation we used anti-FLAG antibodies for cells expressing CENP-A or native CENP-A antibodies. Immunoprecipitation was performed by the previous method (Shang et al., 2010). DNA was extracted from immunoprecipitates and was subjected into a HiSeq 2000 DNA sequencer (Illumina). Sequenced DNAs were mapped into a Chicken Genome database (NCBI, Build 3.1) with a Burrows-Wheeler Aligner (BWA) version 0.6.1 program (Li and Durbin, 2009). We used native CENP-A antibodies for Z#3, #0303, #BM23, #1305, and #1505 (not expressing FLAG-CENP-A).

### FISH and Immunofluorescence

For immunofluorescence analysis, DT40 metaphase spreads were prepared by cytospin method and fixed in 4% paraformaldehyde for 15 min at room temperature. Various rabbit antibodies against chicken centromere proteins were used (see Supplemental Experimental Procedures). Following immunofluorescence analysis, DNA was denatured and FISH was performed with Z-specific satellite marker (Hori et al., 1996) as a probe. All immunofluorescence and FISH images were collected with a Cool SNAP HQ camera (Roper Scientific Japan) mounted on an Olympus IX71 inverted microscope with a 100× objective lens together with a filter wheel. All subsequent analysis and processing of images were performed using MetaMorph software (Molecular Devices Japan).

### DNA Replication Timing

The replication profiling was measured by a protocol that has been described (Hiratani et al., 2008). Detailed procedure is described in Supplemental Information.

Data analyses were done using R/Bioconductor (http://www.r-project.org). To examine the statistical significance of replication timing changes, we first converted data sets to numeric vectors of 9,612 average replication-timing ratios of nonoverlapping 100 kb windows. By SAM, later shifts in replication timing of 100 kb segments at sites of neocentromere formation in both #0514 and #1024 were found to be statistically significant (#0514: p = 1.86 × 10^−5^, q = 0.0153; #1024: p = 7.79 × 10^−3^, q = 0.0903; q value is a false-discovery rate [FDR]-based measure of significance; Storey and Tibshirani, 2003). By stringent criteria, SAM identified 25 genomic segments of 100 kb showing significant changes in #0514 (FDR = 1.8%), which all showed a later shift in replication timing. In #1024, 13 segments showed significant changes (FDR = 1.5%). In BM23, none were identified. The neocentromere formation site in #0514 was among these top-ranked segments, and, in fact, it was the top-ranked segment on chromosome Z.

## Accession Numbers

All ChIP-seq data were deposited to the DNA Data Bank of Japan (DDBJ) under accession number DRA000622.

## Supplemental Information

Document S1. Figures S1–S5 and Supplemental Experimental Procedures
