# Supplementary material for: Chromosome Engineering Allows the Efficient Isolation of Vertebrate Neocentromeres
Source: Dev Cell. 2013 Mar 25;24(6):635–48. doi: 10.1016/j.devcel.2013.02.009 (PMC3925796; doi:10.1016/j.devcel.2013.02.009)
Supplement: Document S1. Figures S1–S5 and Supplemental Experimental Procedures [file mmc1.pdf]

Developmental Cell, Volume *24*

Supplemental Information

Chromosome Engineering Allows the Efficient Isolation of Vertebrate Neocentromeres

Wei-Hao Shang, Tetsuya Hori, Nuno M.C. Martins, Atsushi Toyoda, Sadahiko Misu, Norikazu Monma, Ichiro Hiratani, Kazuhiro Maeshima, Kazuho Ikeo, Asao Fujiyama, Hiroshi Kimura, William C. Earnshaw, and Tatsuo Fukagawa

Inventory of Supplementary Information

Figure S1, A strategy to isolate surviving cells after removal of endogenous Z centromere, related to Figure 1.

Figure S2, Representative images of Immuno-FISH analysis in various types of chromosomes with neocentromere, related to Figure 2.

Figure S3, ChIP-seq analysis in cells with neocentromere, related to Figure 3.

Figure S4, CENP-P or CENP-S is not required for efficient neocentromere formation, related to Figure 3.

Figure S5, Distribution H3K9me3 in DT40 cells, related to Figure 5.

Supplemental Experimental Procedures

Supplemental References

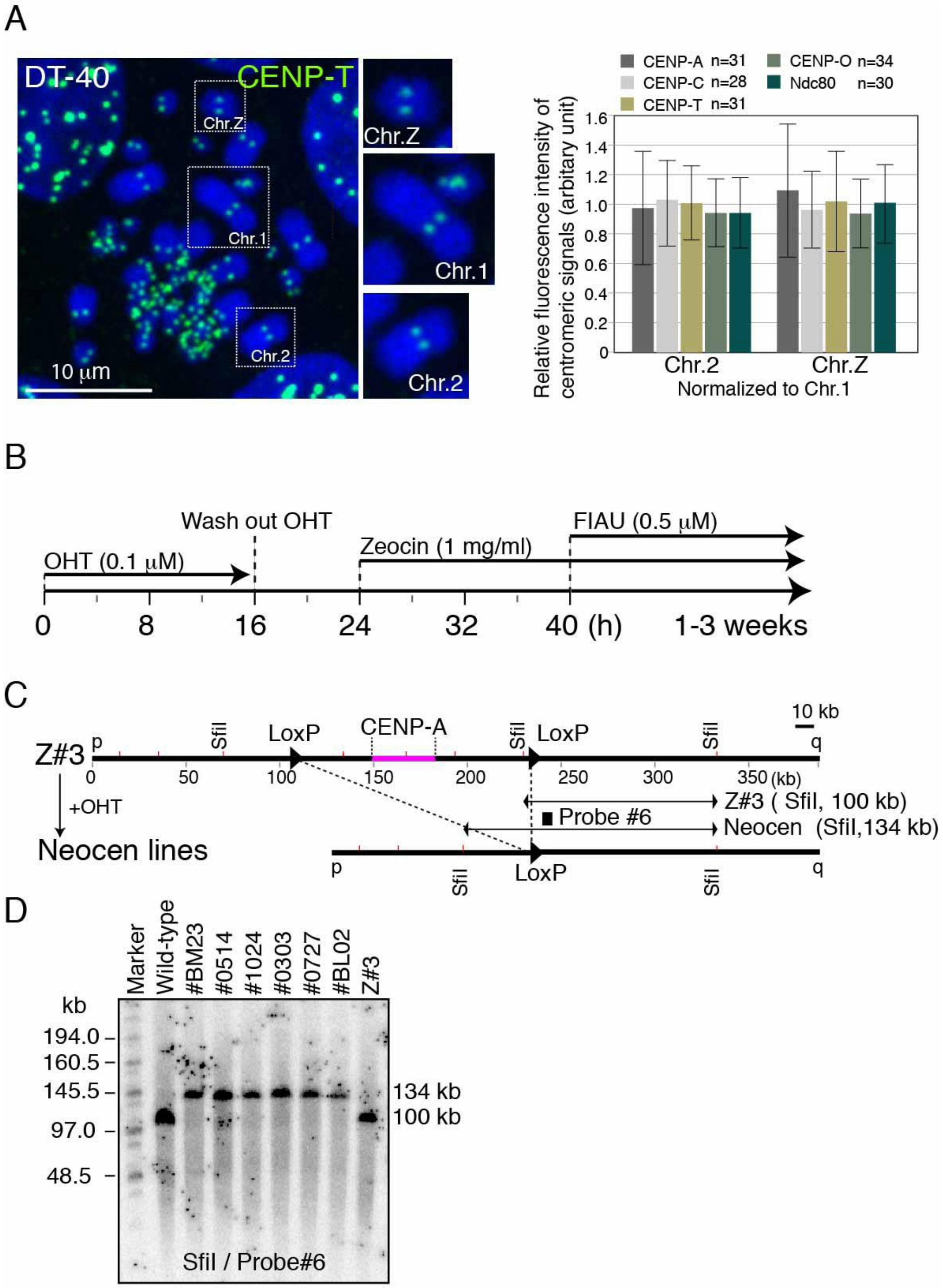
A
DT-40
CENP-T
Chr.Z
Chr.1
Chr.2
10 μm
CENP-A n=31
CENP-C n=28
CENP-T n=31
CENP-O n=34
Ndc80 n=30
Relative fluorescence intensity of centromeric signals (arbitary unit)
Chr.2
Chr.Z
Normalized to Chr.1
B
Wash out OHT
FIAU (0.5 μM)
Zeocin (1 mg/ml)
OHT (0.1 μM)
0
8
16
24
32
40 (h)
1-3 weeks
C
Z#3
p
SfiI
LoxP
CENP-A
SfiI
LoxP
SfiI
10 kb
q
+OHT
Neocen lines
Z#3 (SfiI, 100 kb)
Probe #6
Neocen (SfiI,134 kb)
D
Marker
Wild-type
#BM23
#0514
#1024
#0303
#0727
#BL02
Z#3
kb
194.0
160.5
145.5
97.0
48.5
134 kb
100 kb
SfiI / Probe#6

Figure S1

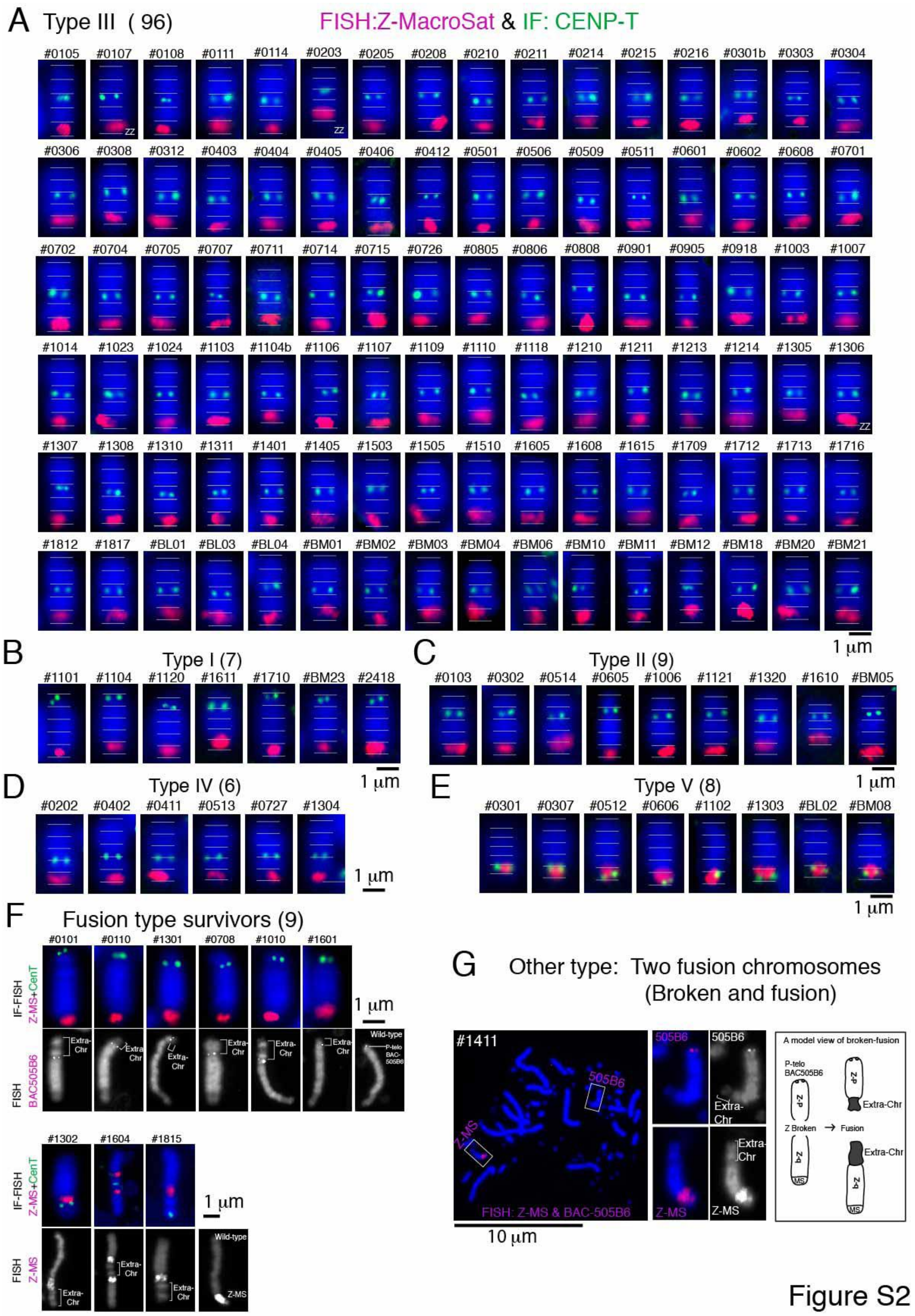
A Type III (96)
FISH:Z-MacroSat & IF: CENP-T
B Type I (7)
C Type II (9)
D Type IV (6)
E Type V (8)
F Fusion type survivors (9)
G Other type: Two fusion chromosomes (Broken and fusion)
#1411
FISH: Z-MS & BAC-505B6
10 μm
A model view of broken-fusion
Extra-Chr
Z Broken → Fusion
1 μm

Figure S2

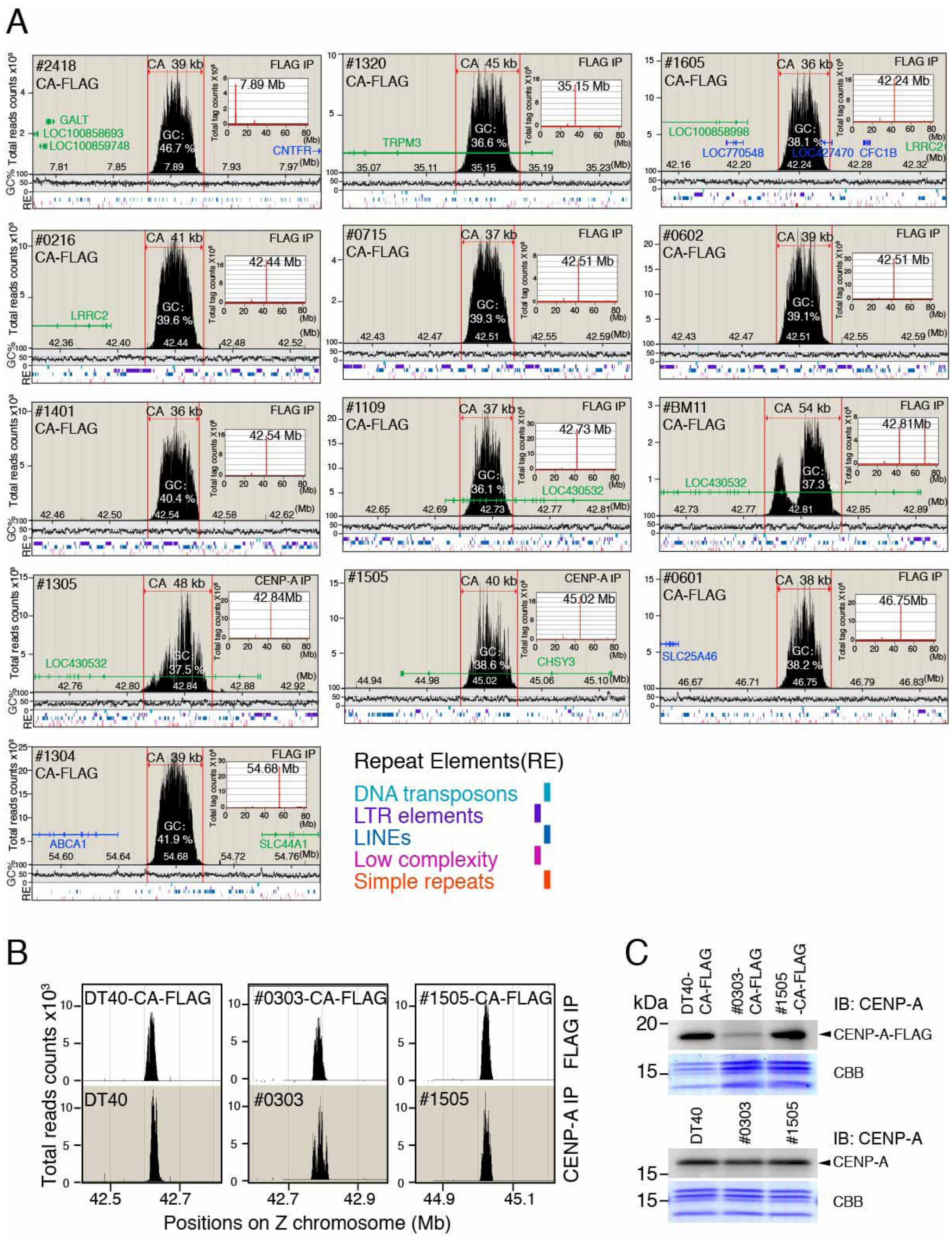

A
#2418 CA-FLAG
CA 39 kb
FLAG IP
7.89 Mb
GC: 46.7 %
#1320 CA-FLAG
CA 45 kb
35.15 Mb
GC: 36.6 %
#1605 CA-FLAG
CA 36 kb
42.24 Mb
GC: 38.1 %
#0216 CA-FLAG
CA 41 kb
42.44 Mb
GC: 39.6 %
#0715 CA-FLAG
CA 37 kb
42.51 Mb
GC: 39.3 %
#0602 CA-FLAG
CA 39 kb
42.51 Mb
GC: 39.1%
#1401 CA-FLAG
CA 36 kb
42.54 Mb
GC: 40.4 %
#1109 CA-FLAG
CA 37 kb
42.73 Mb
GC: 36.1 %
#BM11 CA-FLAG
CA 54 kb
42.81Mb
GC: 37.3 %
#1305
CA 48 kb
CENP-A IP
42.84Mb
GC: 37.5 %
#1505
CA 40 kb
45.02 Mb
GC: 38.6 %
#0601 CA-FLAG
CA 38 kb
46.75Mb
GC: 38.2 %
#1304 CA-FLAG
CA 39 kb
54.68 Mb
GC: 41.9 %
Total reads counts x10³
Total tag counts X10⁶
GC%
RE
Repeat Elements(RE)
DNA transposons
LTR elements
LINEs
Low complexity
Simple repeats
B
DT40-CA-FLAG
#0303-CA-FLAG
#1505-CA-FLAG
DT40
#0303
#1505
FLAG IP
CENP-A IP
Total reads counts x10³
Positions on Z chromosome (Mb)
C
kDa
DT40-CA-FLAG
#0303-CA-FLAG
#1505-CA-FLAG
IB: CENP-A
CENP-A-FLAG
CBB
DT40
#0303
#1505
CENP-A

Figure S3

A

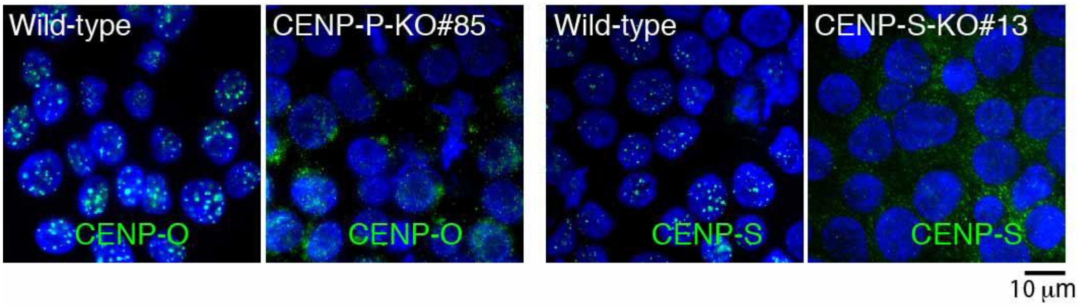

B

	Zcen (-) / 1,000,000 Cells	
Trials	Z#3	P-KO#85
#1	3.0	5.9
#2	4.0	5.1
#3	4.6	4.4
Avg ± SD	3.9 ± 0.8	5.1 ± 0.8

	Zcen (-) / 1,000,000 Cells	
Trials	Z#3	S-KO#13
#1	2.3	2.2
#2	3.4	2.7
#3	3.8	3.7
Avg ± SD	3.2 ± 0.8	2.9 ± 0.8

Figure S4

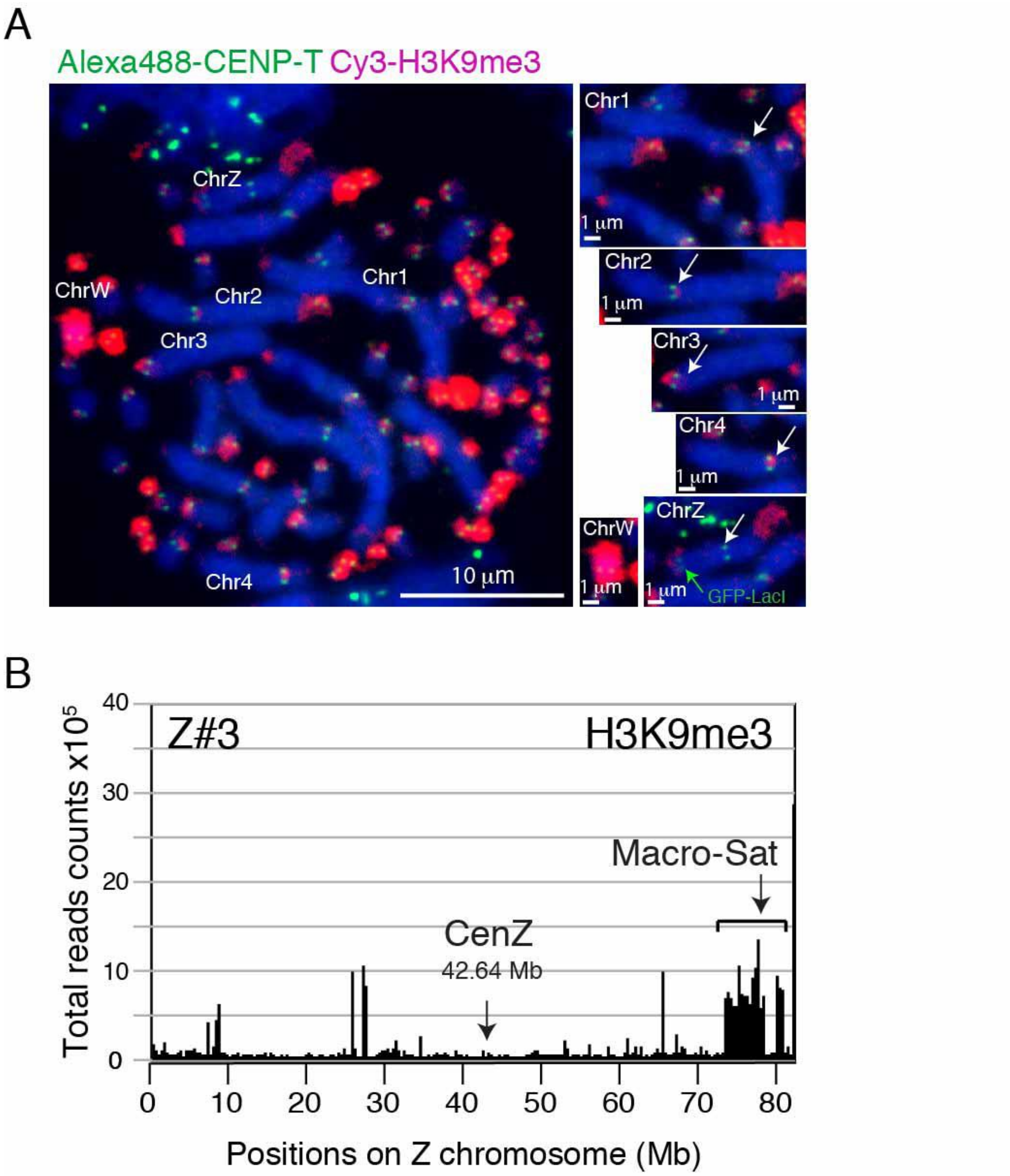
A
Alexa488-CENP-T Cy3-H3K9me3
ChrZ
ChrW
Chr2
Chr1
Chr3
Chr4
10 μm
Chr1
1 μm
Chr2
1 μm
Chr3
1 μm
Chr4
1 μm
ChrW
1 μm
ChrZ
1 μm
GFP-LacI
B
Z#3
H3K9me3
Macro-Sat
CenZ
42.64 Mb
Total reads counts x10^5
0
10
20
30
40
Positions on Z chromosome (Mb)
0
10
20
30
40
50
60
70
80

Figure S5

Legends for Supplementary Figures

Figure S1 A strategy to isolate surviving cells after removal of endogenous Z centromere, related to Figure 1.

(A) Measurement of signal intensities of anti-CENP-A, CENP-C, CENP-T, CENP-O, and Ndc80 on chromosome 1, 2, and Z. A typical image for CENP-T staining on chicken chromosome spread was shown. These signal intensities were similar between chromosome 1, 2, and Z. Error bars show standard deviation (SD).

(B) Experimental time-line to isolate neocentromere-containing cells. OHT was added to activate Cre-recombinase and cells were cultured for 16 h and OHT was washed out at 16 h. Zeocin was added for positive selection at 24 h after the addition of OHT and FIAU was added for negative selection at 40 h after the addition of OHT. Surviving colonies were isolated 1-3 weeks later after the OHT addition.

(C) Probe information (probe #6) for Southern analysis shown in Figure S1D.

(D) High molecular weight genome DNAs extracted from various cell lines with neocentromere were digested with Sfi I and were applied to pulsed-filed gel electrophoresis. A gel was transferred and was hybridized with the probe #6.

Figure S2 Representative images of Immuno-FISH analysis in various types of chromosomes with neocentromere, related to Figure 2.

(A) Images of Type III chromosome (96 examples). Red shows satellite sequence on q-arm of chromosome Z. Centromeres (green) are visualized by anti-CENP-T antibodies.

(B) Images of Type I chromosome (7 examples).

(C) Images of Type II chromosome (9 examples).

(D) Images of Type IV chromosome (6 examples).

(E) Images of Type V chromosome (8 examples).

(F) Images of fusion chromosome Z with another chromosome. BAC505B6 containing p-telomere region was used as a probe to confirm p-arm fusion. Six cases were p-arm fusion. Three cases were q-arm fusion.

(G) A cell line (#1411) contains two fusion chromosomes. Each fusion chromosome contains p-arm and q-arm of Z chromosome, respectively. Our model is shown in right

cartoon: chromosome Z was broken in middle region and p-arm and q-arm were independently fused with another chromosome and two fusion chromosomes were generated.

Figure S3 ChIP-seq analysis in cells with neocentromere, related to Figure 3.

(A) ChIP-seq analysis with anti-FLAG or anti-CENP-A antibodies in cells with neocentromere. We used anti-FLAG antibodies for cells expressing CENP-A-FLAG (FLAG-IP). For cells not expressing CENP-A-FLAG, we used native CENP-A antibodies (CENP-A-IP). IP DNAs were deeply sequenced and sequence data were mapped on chicken genome database. We firstly identified a major peak as a neocentromere from entire chromosome Z in each cell line and examined detail distribution around the peak with a higher resolution. GC% contents and distribution of transposons, repeat sequence, and genes are shown as in Figure3.

(B) Comparison of ChIP-seq profiles with anti-CENP-A of cells not expressing CENP-A-FLAG with cells expressing CENP-A-FLAG.

(C) Expression level of CENP-A-FLAG in DT40-CA-FLAG, #03030-CA-FLAG and #1505-CA-FLAG cells. CENP-A-FLAG was not over expressed in these cell lines.

Figure S4 CENP-P or CENP-S is not required for efficient neocentromere formation, related to Figure 3.

(A) Immunofluorescence analysis of wild-type DT40 or CENP-P-deficient cells with anti-CENP-O antibodies (left two panales) and immunofluorescence analysis of wild-type DT40 or CENP-S-deficient cells with anti-CENP-S antibodies (right two panels).

(B) Numbers of cells with neocentromere. We independently performed 3 trials for this assay.

Figure S5. Distribution H3K9me3 in DT40 cells, related to Figure 5.

(A) Immunofluorescence analysis on mitotic chromosome spread from chicken DT40 cells with Alexa488-labeled-anti-CENP-T (green) and Cy3-labeled-anti-H3K9me3 (red). H3K9me3 signals were not detected in centromere of chromosome Z, but were detected in centromeres of chromosome 1, 2, 3, and 4, which contain tandem-repetitive sequences. We note that as repetitive region on centromere is long (~Mb) and

kinetochore domain is short (30-40-kb), H3K9me3 signals did not overlapped wirh CENP-T signals on repetitive centromeres. Entire region of chromosome W is highly heterochromatinized. Strong accumulation of H3K9me3 in entire chromosome W was observed.

(B) Genome wide ChIP-seq profile with anti-H3K9me3 on chromosome Z in DT40 cells. H3K9me3 was not enriched around centromere region, but accumulated in macro-satellite region on the q-arm in chromosome Z. This data is consistent with immunofluorescence shown in (A).

Supplemental Experimental Procedures

DNA replication timing (detailed method)

BrdU labeling time was 1 h. Sample labeling, microarray hybridization and data extraction were performed according to standard procedures by NimbleGen using a chicken whole-genome microarray with one probe every 2.6 kb (Roche NimbleGen Inc., galGal3 WG CGH; 385K oligonucleotide probes). For all samples, two independent biological replicates were analyzed, for which early- and late-replicating DNA were labeled reciprocally with Cy3 and Cy5 (=dye switch). In every case, replicates of the same cell line showed very high correlation, verifying the reproducibility and stability of profiles for a given cell line. All data sets are graphically displayed and are downloadable at: http://www.replicationdomain.org

Data analyses were done using R/Bioconductor (http://www.r-project.org). To calculate average replication timing of 100-kb segments, replication-timing ratios of non-overlapping 39 neighboring probes were averaged (39 x 2.586 kb spacing = 100.854 kb). To run SAM (Significance Analysis of Microarrays), we used an R package, siggenes (http://www.bioconductor.org/packages/2.11/bioc/html/siggenes.html). To examine statistical significance of replication timing changes, we first converted data sets to numeric vectors of 9,612 average replication-timing ratios of non-overlapping 100-kb windows. By SAM, later shift in replication timing of 100-kb segments at sites of neocentromere formation in both #0514 and #1024 were found to be statistically significant [#0514: p value=1.86E–5, q value=0.0153; #1024: p value=7.79E–3, q value=0.0903; q value is a FDR (false discovery rate)-based measure of significance (Storey and Tibshirani, 2003)]. By stringent criteria, SAM identified 25 genomic segments of 100-kb showing significant changes in #0514 (FDR=1.8%), which all showed a later shift in replication timing. In #1024, 13 segments showed significant changes (FDR=1.5%). In BM23, none were identified. The neocentromere formation site in #0514 was among these top ranked segments, and in fact it was the top ranked segment on chromosome Z.

Antibodies

Centromere antibodies used in this study are following: anti-CENP-A (a rabbit antibody against a synthetic peptide for chicken CENP-A 2-15 aa), anti-CENP-C (a rabbit antibody against recombinant chicken CENP-C 1-330 aa), anti-CENP-E (a rabbit antibody against recombinant chicken CENP-E 565-1088 aa), anti-CENP-O (a rabbit antibody against recombinant chicken full-length CENP-O), anti-CENP-T (a rabbit antibody against recombinant chicken full-length CENP-T), anti-Ndc80 (a rabbit antibody against recombinant chicken Ndc80 465-640 aa), anti-KNL2 (a rabbit antibody against recombinant chicken KNL2 1081-1501 aa), anti-Aurora B antibodies (a rabbit antibody against recombinant chicken full-length Aurora B). Some of these centromere antibodies were used in previous studies (Hori et al., 2008; Kline et al., 2006; Okada et al., 2006). Monoclonal antibodies for histone modification including H3K9me3, H3K4me2 and H3K36me3 were created by H. Kimura (Kimura et al., 2008).

Supplemental References

Hori, T., Amano, M., Suzuki, A., Backer, C.B., Welburn, J.P., Dong, Y., McEwen, B.F., Shang, W.H., Suzuki, E., Okawa, K., *et al.* (2008). CCAN makes multiple contacts with centromeric DNA to provide distinct pathways to the outer kinetochore. Cell *135*, 1039-1052.

Kimura, H., Hayashi-Takanaka, Y., Goto, Y., Takizawa, N., and Nozaki, N. (2008). The organization of histone H3 modifications as revealed by a panel of specific monoclonal antibodies. Cell structure and function *33*, 61-73.

Kline, S.L., Cheeseman, I.M., Hori, T., Fukagawa, T., and Desai, A. (2006). The human Mis12 complex is required for kinetochore assembly and proper chromosome segregation. The Journal of cell biology *173*, 9-17.

Okada, M., Cheeseman, I.M., Hori, T., Okawa, K., McLeod, I.X., Yates, J.R., 3rd, Desai, A., and Fukagawa, T. (2006). The CENP-H-I complex is required for the efficient incorporation of newly synthesized CENP-A into centromeres. Nature cell biology *8*, 446-457.

Storey, J.D., and Tibshirani, R. (2003). Statistical significance for genomewide studies. Proceedings of the National Academy of Sciences of the United States of America *100*, 9440-9445.
